# Mr. Leeson, on Cataract

**Published:** 1802-12-01

**Authors:** B. Leeson

**Affiliations:** Member of the Royal College of Surgeons. Grantham


### *To the Editors of the Medical and Phyſical Journal.*

GENTLEMEN,

IN your Journal for May, I obſerved an account of a caſe of Cataract, in which the accidental occurrence of inflammation had removed the diſeaſe. From which, together with ſome obſervations of Mr. Ware's, the ingenious narrator, Mr. Crowfoot, ſeems to think, that it might be ſometimes expedient to excite artificial inflammation in the tunica conjunctiva or cornea, for the removal of that diſeaſe. The two following caſes may probably give ſome idea of the ſucceſs to be expected from ſuch practice; and are therefore much at your ſervice, if you eſteem them worth inſerting in your very uſeful publication.

I am, &c.

*Grantham, Oct.* 17, 1802.

B. LEESON, Jun.
Member of the Royal College of Surgeons.

---

ROBERT SMITH has been blind of his left eye from his infancy, which defect evidently aroſe from a collection of opaque, milky fluid, within the capſule of the cryſtalline lens, occaſioning an unuſual prominence of that eye. He is a very ſtout, athletic man, about three and twenty years of age, conſtantly employed in the occupations of huſbandry, and very regular in his mode of life. On the eleventh of March, he applied to me in conſequence of violent inflammation, which had attacked the diſeaſed difeafed eye, attended with very fevere pain in his head, and very general increafed action in the fyftem. The ufual antiphlogiftic method of treatment was purfued, together with fcarifications of the tunica conjunctiva, bleeding from the temporal artery, and the application of the vinum thebaicum to the eye. In about ten days the inflammation was fubdued. It now became defirable to afcertain what effect the inflammation would produce on the previous difeafe, and the eye has fince been infpected at different times for that purpofe. When a month had elapfed from the ceffation of the inflammation, the cryftalline lens was obferved to have become perfectly clear, and has ever fince remained fo; the globe of the eye is alfo leffened, but ftill the vifion is equally imperfect, as before this attack of inflammation; although no other appearance of difeafe is evident, than a leffened fenfibility in the iris to the ftimulus of light.

Elizabeth Rimmington, aged twenty-one, is perfectly well in health, of a firm, mufcular habit; has cataracts in both eyes, which have been there fince her infancy. She has fufficient vifion to diftinguifh light from darknefs, and to guide herfelf in places to which fhe is accumftomed. The cataracts have the appearance of being in a foft ftate, giving the idea of a milky fluid being enclofed within the capfule. As the left eye was poffeffed of moft vifion, it was propofed to operate firft on the right eye, and fhould that fucceed, to proceed to the other at fome future period.

On the 20th of December, 1801, having prepared for the operation, a knife was paffed through the cornea in the ufual manner, and the capfule of the cryftalline being wounded, a milky fluid was immediately evacuated with fome force, part being expelled through the incifion, and part effufed through the globe of the eye. Under thefe circumftances, it was unneceffary to complete the incifion, and I therefore withdrew the knife. The patient was put to bed in a dark room, and treated in the ufual manner; for feveral days I had the fatisfaction to believe, that fhe would recover the fight of that eye with little pain. The inflammation and fever fucceeding the operation being trifling, at the end of a week fhe was releafed from her confinement. At this time, the effufed liquor was perfectly abforbed, and the eye was, in every appearance, free from difeafe, the iris contracting and dilating very rapidly on the admiffion or expulfion of light; ftill her vifion was lefs perfect than immediately after the operation. Apprehending this to arife from the capfule remaining unabforbed, I recommended nothing to be applied for a fhort time, hoping abforption would come on, as the office of the part was now no longer neceffary. This not taking place in a month, fome ftimulating applications, with a view to promote abforption, were ufed, but without any

 better better effect; and I am sorry to add, that at this time, October the 14th, the patient remains with the same imperfect vision as before the operation, notwithstanding the eye is free from any visible imperfection. In the first of these cases, the diminished sensibility of the iris may shew some deficiency in the optic nerve, probably co-existent with the formation of the original disease. But in the second, the eye exhibits every appearance of health, and it is only upon the opinion of the non-absorption of the crystalline capsule, that the present blindness is to be accounted for, and even this might be supposed to occasion some visible defect in the eye. This supposition contradicts the opinion of the best modern surgeons, who maintain that parts will continue no longer than their functions are necessary, on which opinion, the operation for the extraction of the cataract is supported. As far therefore as this case goes, it will lead to a very guarded prognosis in every operation, even in the usual mode, for cataract; and to a much more guarded one, should the mode of treatment suggested by Mr. Crowfoot be adopted.

